# Reading immune restoration through *Mycobacterium tuberculosis* intrahost evolution

**DOI:** 10.1128/msystems.00953-26

**Published:** 2026-09-22

**Authors:** Lin Teng, Guangzhi Zhang

**Affiliations:** 1 Key Laboratory of Systems Health Science of Zhejiang Province, School of Life Science, Hangzhou Institute for Advanced Study, University of Chinese Academy of Sciences 638898, Hangzhou, China; 2 Institute of Animal Science, Chinese Academy of Agricultural Sciences 12661, Beijing, China; School of Life Science, Hangzhou Institute for Advanced Study, Hangzhou, Zhejiang, China

**Keywords:** *Mycobacterium tuberculosis*, intrahost evolution, HIV, pathogen genomics, immune restoration

## Abstract

Antiretroviral therapy (ART) suppresses human immunodeficiency virus replication, but does not fully restore protection against tuberculosis. The relevant mechanisms are still poorly understood. M. C. Chao, M. R. Chase, S. Wakabayashi, A. J. Vickers, et al. (mSystems 11:e00329-26, 2026, https://doi.org/10.1128/msystems.00329-26) use population-level *Mycobacterium tuberculosis* genome sequencing in macaques to show that similar bacterial burdens can conceal different evolutionary trajectories: simian immunodeficiency virus coinfection primarily increases opportunities for mutation through replication, whereas ART-treated animals show a distinct enrichment of oxidative damage-associated variants. We argue that the principal value of this work is conceptual. Pathogen genomes may provide a time-integrated, but indirect, readout of local immune ecology. To translate these genomic signatures into a framework for evaluating immune restoration, future studies should pair host and pathogen measurements, experimentally validate the mechanisms generating these mutations, and longitudinally link tissue-level selection to persistence, reactivation, and transmission.

## COMMENTARY

## BEYOND VIRAL SUPPRESSION

A recent study by Chao et al. addresses a question that conventional treatment endpoints cannot answer: after HIV replication is suppressed, has the tissue environment that constrains *Mycobacterium tuberculosis* (Mtb) actually returned to normal (1)? This distinction matters because people receiving long-term antiretroviral therapy (ART) retain excess tuberculosis risk, alveolar macrophages can show incomplete epigenetic responses to Mtb, and suppressive ART did not prevent extrapulmonary dissemination in a macaque model (2–4). Viral load therefore measures control of HIV, but not necessarily restoration of the local immune ecology encountered by Mtb.

## WHAT POPULATION GENOMICS ADDS

Chao et al. sequenced Mtb populations from 477 tissues across 20 macaques and combined newly acquired variants with pre-existing bacterial barcodes (1, 5). The resulting analysis refines dissemination networks and separates two processes that are easily conflated. Simian immunodeficiency virus coinfection increased the number of observed mutations largely by permitting more bacterial replication, not by detectably increasing the mutation rate per generation. ART-treated animals, by contrast, showed a different composition of mutations, including an enrichment trend for oxidative damage-associated changes. Thus, mutation abundance and mutational spectrum report different biological dimensions: the opportunity to diversify and the conditions under which diversification occurs. This distinction is more informative than treating every additional variant as evidence of stronger mutagenesis or adaptation (6).

The secondary-barcode strategy also changes how dissemination can be interpreted. A synthetic barcode identifies a founding lineage, whereas an acquired mutation can subdivide that lineage and suggest an order of spread. Yet, directionality remains inferred rather than observed. Variants can be missed when rare, lost through bottlenecks, or detected only in tissues sampled at necropsy. The networks are therefore constrained evolutionary reconstructions, not complete maps of bacterial movement.

## A RECORDER BUT NOT A READOUT

**Fig 1:**
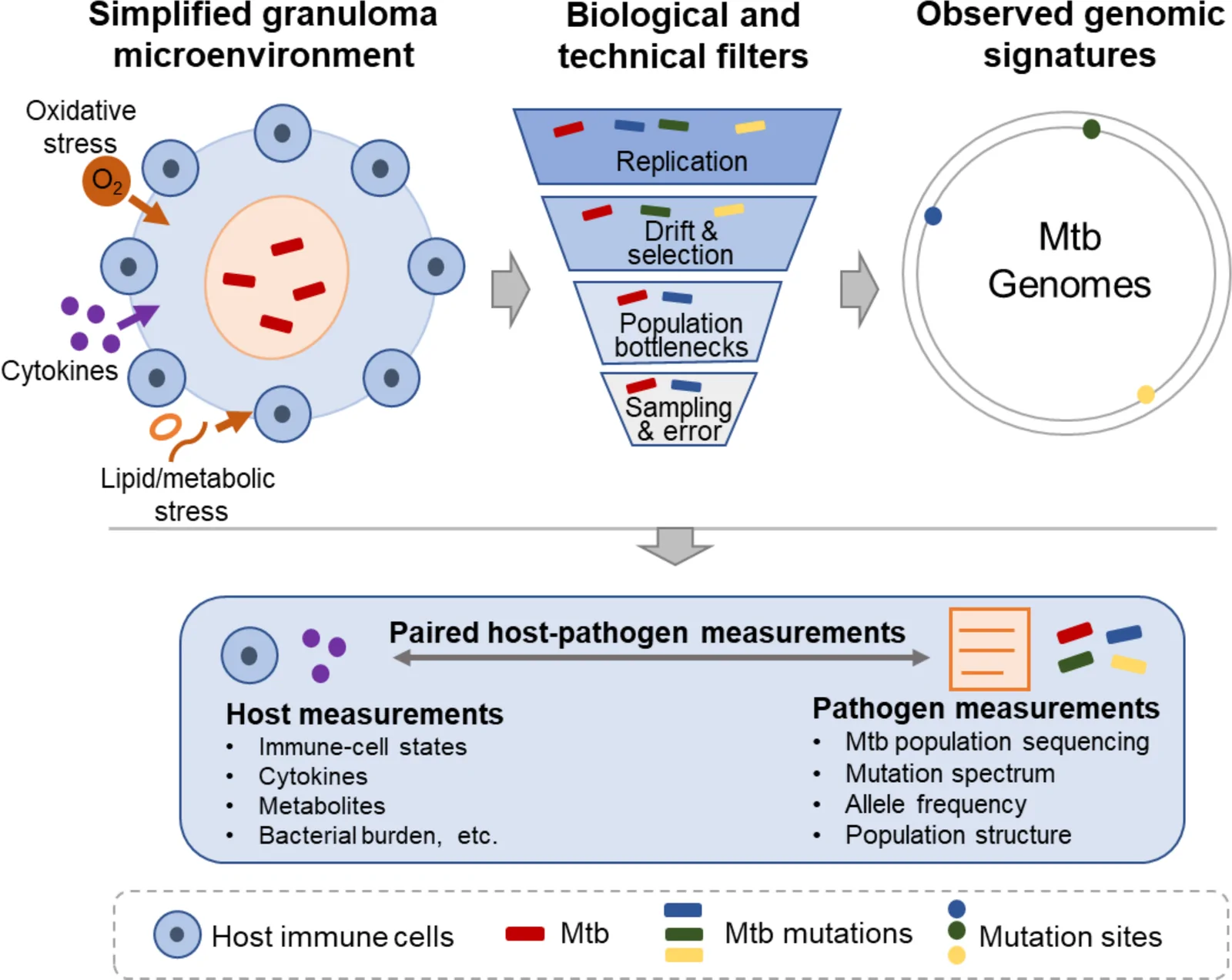
Host microenvironmental pressures are indirectly reflected in *Mycobacterium tuberculosis* genomic variation. Oxidative conditions, cytokine signaling, and the local lipid and metabolic environment within spatially heterogeneous granulomas may influence the generation and fate of intrahost Mtb variants. However, the genomic signatures ultimately detected by population sequencing are further shaped by bacterial replication, genetic drift, and selection, population bottlenecks, tissue sampling, and technical error. Observed variants therefore represent time-integrated but indirect traces of the host microenvironment rather than one-to-one readouts of specific immune pathways. Paired profiling of host immune-cell states, cytokines, metabolites, bacterial burden, and Mtb population genomics within the same lesion will be required to distinguish immune-associated mutational processes from alternative biological and technical explanations.

The useful metaphor of the pathogen genome as a molecular recorder requires qualification. A mutation is a historical trace filtered by bacterial replication, population bottlenecks, genetic drift, selection, sampling, and technical error (Fig. 1). It is not a one-to-one readout of cytokine activitie or immune pathway functions. In the present study, oxidative mutations were associated with lower interleukin-10 (IL-10) expression in myeloid cells and lower bacterial burdens at a limited number of profiled lesions (1). Prior work independently links host immune environments to Mtb mutational spectra (7) and shows that IL-10 can inhibit phagosome maturation in infected macrophages (8). These observations are consistent with stronger local immune pressure, but they do not establish that reduced IL-10 increased reactive oxygen species or directly caused the mutations. The treatment-group enrichment was also a trend, and oxidative damage can arise during DNA preparation. The strongest interpretation is therefore that ART-treated hosts may impose a distinct set of microenvironmental pressures despite similar aggregate bacterial outcomes.

A useful recorder must also be calibrated against time and exposure. Identical spectra could arise from brief intense stress or weaker stress acting across more bacterial generations, while different spectra could reflect lineage-specific DNA repair rather than different host responses. Future analyses should therefore normalize for replication history, include extraction controls, and compare lesions within the same animal whenever possible. Such calibration would convert a compelling metaphor into a falsifiable measurement framework.

## FROM RECURRENT TARGETS TO TESTABLE MECHANISMS

The enrichment of mutations in lipid metabolism and polyketide synthase genes provides a second opportunity for interpretation but not yet proof of adaptive convergence. Mtb adapts to the intracellular environment through tightly regulated metabolic and redox responses, including the sensing of O_2_ and nitric oxide and the utilization of host-derived cholesterol (9, 10). Recurrent variation in these pathways could reflect positive selection, relaxed constraint, mutational target size, or hitchhiking. Resolving these alternatives will require reconstructing individual alleles and testing their effects under defined carbon sources, redox conditions, macrophage states, and transmission bottlenecks. The relevant question is not simply whether the same gene mutates repeatedly, but which allele changes bacterial fitness in the niche where it arose. This allele-centered approach would clarify whether variants that expand locally are also likely to disseminate or transmit. Local fitness, tissue escape, persistence during treatment, and between-host transmission impose different filters. A mutation favored in one lesion may be neutral or costly at the next scale.

## OUTLOOK: MEASURING THE QUALITY OF IMMUNE RESTORATION

This work suggests a forward-looking framework in which treatment success is evaluated across host, lesion, pathogen, and population scales (Fig. 1). Three tests are especially important. First, spatially paired measurements should link Mtb variants to local reactive oxygen species, cytokines, metabolites, immune-cell states, bacterial burden, and DNA-damage controls in the same lesion. Second, longitudinal or staged sampling should determine whether early signatures persist through latency, reactivation, treatment, and dissemination; human autopsy and serial sputum studies show both the value and the sampling limits of such inference (6, 11). Third, interventions should be tested prospectively. If a vaccine or host-directed therapy changes immune pressure, investigators should predefine the expected host measurements and mutational signatures, then ask whether both change concordantly. Genomics alone should not be used as a surrogate endpoint until that relationship is validated.

A key challenge for extending these findings to humans is that sputum-derived Mtb may not fully represent the bacterial populations and selective pressures within individual granulomas. Airway samples may capture dominant pulmonary populations while missing lymph node or extrapulmonary niches. Combining serial sputum sequencing with imaging, blood immune profiling, and clinically indicated tissue sampling could test whether accessible samples predict spatially hidden disease. The goal is not to replace host measurements, but to identify discordance between systemic recovery and the microbial experience within lesions. The broader contribution of Chao et al. (1) is therefore not a claim that Mtb genomes reveal immune state directly. It is the demonstration that equivalent clinical or microbiological endpoints can conceal different evolutionary histories. Coupling those histories to contemporaneous host measurements could reveal whether ART restores merely systemic viral control or also the tissue-level conditions required for durable control of tuberculosis.
